# A Rare Case of Femoral Neck Fracture in a Six-Year-Old Girl

**DOI:** 10.7759/cureus.17373

**Published:** 2021-08-23

**Authors:** Seddig M Fallatah, Yaquob A Daghriri, Amgad A Afifi, Faisal A Alghamdi, Ayman F Zein

**Affiliations:** 1 Orthopedic Surgery, King Salman Armed Forces Hospital, Tabuk, SAU

**Keywords:** fracture neck of femur, pediatric, delbet classification, smith peterson approach, hip spica application

## Abstract

Proximal femur fractures are rare pediatric injuries associated with high energy trauma as well as polytrauma. Injuries during childhood can cause a significant disability in some cases. The four Delbet classifications of femur fractures are frequently used as prognostic for potential avascular necrosis. Necessary treatment is urgent and needs to be tailored to the fracture type and patient age. This case report presents the detailed history, examination, and treatment of a six-year-old girl with an uncommon site of pediatric fracture at the femoral neck combined with an ipselateral displaced talus fracture due to a fall from the second floor. Talus fracture was missed and discovered in operation room; however, both fractures are highly associated with avascular necrosis and post-traumatic arthritis. The patient was followed for two years on regular basis after the initial operation with a normal gait, full range of motion, and no active complain.

## Introduction

Pediatric femoral neck fractures are rare, frequently caused by a high mechanism of injury, and commonly combined with multiple trauma [1-4]. It is also associated with a risk of possible long-term dysfunction and adverse complications [5-7]. In Saudi Arabia, a study of 1456 diagnosed cases of accidental fractures and dislocations in children was carried out to assess the patterns of fractures and dislocations [8]. The result showed that the most commonly stumble-on fractures were the forearm (39.6%), humerus (12%), clavicle (11%), tibia and fibula (10.6%), and femur (5.6%). The male-to-female ratio was 2.3:1, and the risk of injury appeared to be higher in preschool children (two to six years) than in adolescents (12-14 years). Non-road traffic accidents were the cause of 93% of all cases.

Proximal femur fracture in pediatrics accounts for 1.2 to two cases per year, which is considered 0.3-0.5% of fractures in children [1-4]. The peak incidence rate is between the age of 10 years and 13 years old (total range: one day to 18 years), with a 1.3-1.7:1 male-to-female ratio [3,5,6]. Dysfunction and pain are the most frequently reported complications in 20-50% of all patients. These complications are due to osteonecrosis, coxa valga, proximal femoral physeal growth arrest, and non-union [1-3,7-10].

Approximately, half of all proximal femur fractures in pediatrics are the result of the high mechanism of injury, such as a road traffic accident, and can be related to serious injuries, consisting of injury to the head, chest or abdomen, pelvic ring injury, acetabular fracture, hip dislocation, and ipsilateral femur fracture [1-3,5,6,9,11-15]. Therefore, specific attention must be paid in collaboration with general surgery and neurosurgery to identify other associated injuries, specifically non-musculoskeletal injuries. Open injuries and neurovascular status must be evaluated.

Classification of proximal femur fractures in pediatrics

Delbet classification of proximal femoral fractures, which is a fracture classification guide now regularly used to educate patients on the risks of possible complications prior to initiating treatment. Type I fractures are transphyseal, while types II, III, and IV are transcervical, cervicotrochanteric, and intertrochanteric fractures, respectively. This anatomic fracture classification is prognostic of long-term outcomes as well as the main complication of pediatric femoral neck fractures, osteonecrosis [1,2,8,16]. Osteonecrosis occurs in 16-47% of pediatric proximal femoral fractures [1,2]. And is secondary to a disruption of the vascular supply to the femoral head. Ratliff classified acute osteonecrosis of the femoral head and neck as radiographic sclerosis and collapse of the head (type I), focal sclerosis superior lateral head (type II), or sub-capital neck (type III) with preservation of the epiphyseal supply [5,17,18]. Many authors report that the long-term outcomes of management of Delbet type I fractures are worse when compared to other Delbet type fractures [1,2,6,8,15]. Sub-capital or Salter-Harris type I fractures with complete dislocation of the epiphysis (i.e., Delbet type IB) are universally thought to progress to osteonecrosis regardless of treatment. Debate exists on whether radiographic evidence of sclerotic changes associated with Ratliff type III fracture reflects osteonecrosis rather than routine fracture healing.

## Case presentation

**Figure 1:**
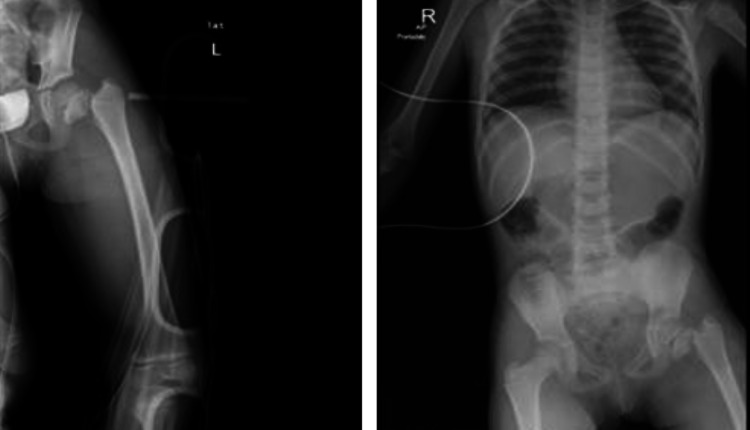
X-ray of left hip and pelvis

**Figure 2:**
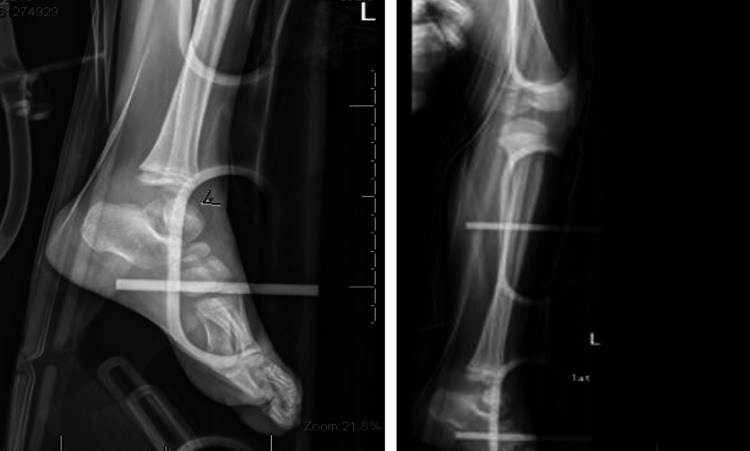
X-ray of left ankle and leg

A six-year-old girl was brought to the emergency room after falling from the second floor when playing at home. She is not known to have any chronic medical illness. She could not stand or bear her body weight when standing and complained of left hip pain. The Advanced Trauma Life Support (ATLS) protocol was completed. She was conscious and alert with normal and stable vital signs. A full head-to-toe examination showed superficial bruising over the dorsal aspect of the left foot. Locally, there was tenderness over the left hip on deep palpation associated with lateral and posterior bruises, but there were no open wounds or visible swelling. Her range of motion of the left hip was restricted due to pain. Radiographs of the pelvis showed a type I transphyseal fracture without epiphyseal dislocation and a type III cervicotrochanteric ipsilateral femoral neck fracture (Figure 1). An ankle x-ray showed a displaced talus fracture (Figure 2).

**Figure 3:**
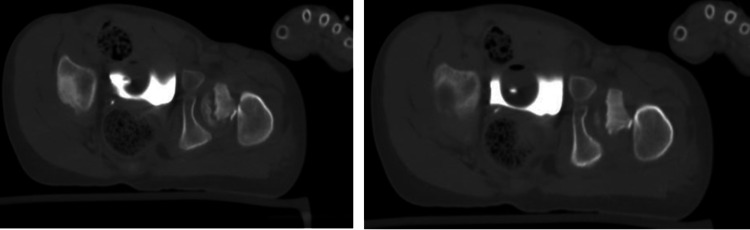
CT scan of the pelvis shows neck of femur fracture

**Figure 4:**
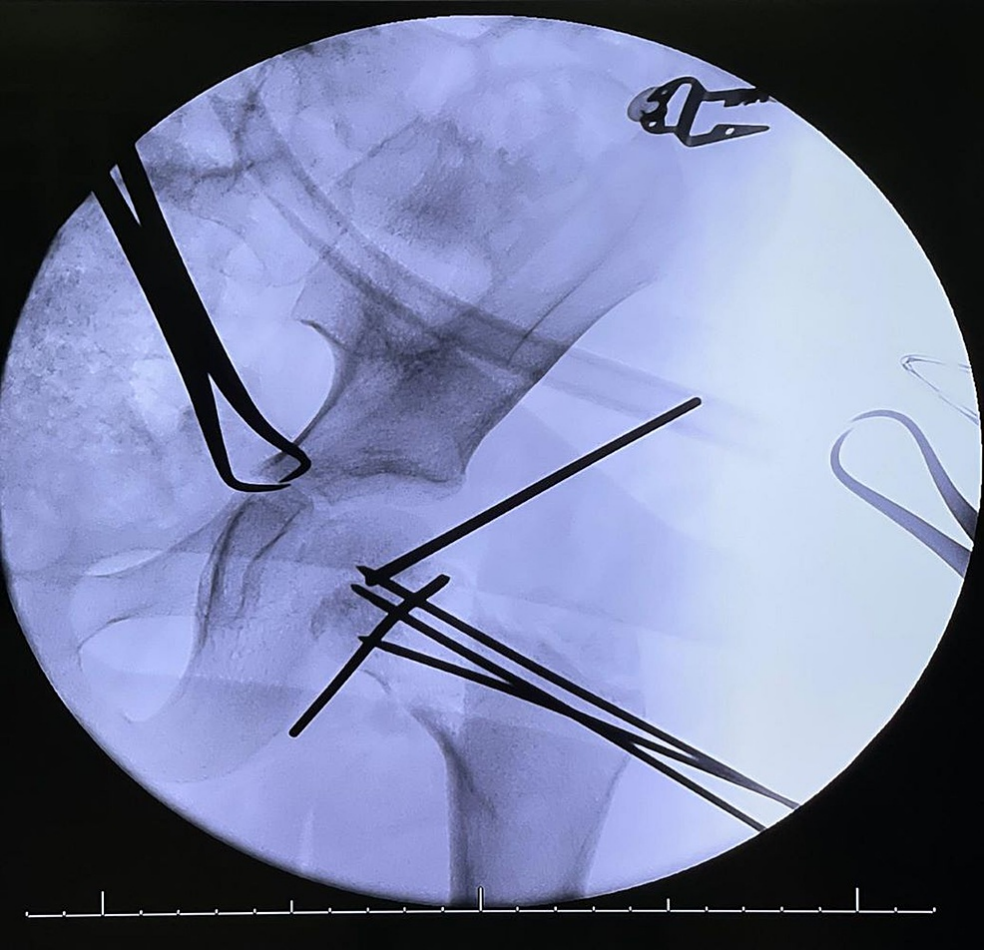
Intraoperative evaluation of the neck of femur reduction procedure by image intensifier

After studying the x-rays and CT scans for the pelvis, treatment options were discussed with the patient's family (Figure 3). The patient underwent a clinical assessment by an anesthesiologist for an urgent operative intervention to fix the fracture. The patient was taken to the operative room and placed in the supine position under general anesthesia with a full aseptic technique. An image intensifier was used as an intraoperative assessment of the fracture reduction and implant placement. A trial of closed reduction failed, thus the surgical team moved to an open reduction technique. Between the sartorius and tensor fasciae latae, the anterior hip approach was performed to reach the capsule of the hip joint and the dissection was made throughout the layers. The hip joint was opened in a T-shape, the fracture lines and three parts were identified as follows: (1) femoral head, (2) slippage physical injury, and (3) neck of femur fracture. Then, the team reduced the fracture using five k-wires: the first holding femoral head with slippage physis, which was rotated; the second holding the epiphysis with the neck as a joystick; and then three additional k-wires were inserted parallel through the neck and holding the neck, physis, and femoral head. As the final step of hip reduction, the surgical team evaluated the reduction under a C-arm then fixation was done using three cannulated screws, closed the capsule with absorbable sutures, and sterile dressing was applied (Figure 4). However, the fixation process was supported by hip spica as a cast immobilization. Additionally, the addressing of ipsilateral talus fracture trail closed has been achieved and insertion of two partially threaded screws posterior to anterior was done.

Post-operative management and follow-up

**Figure 5:**
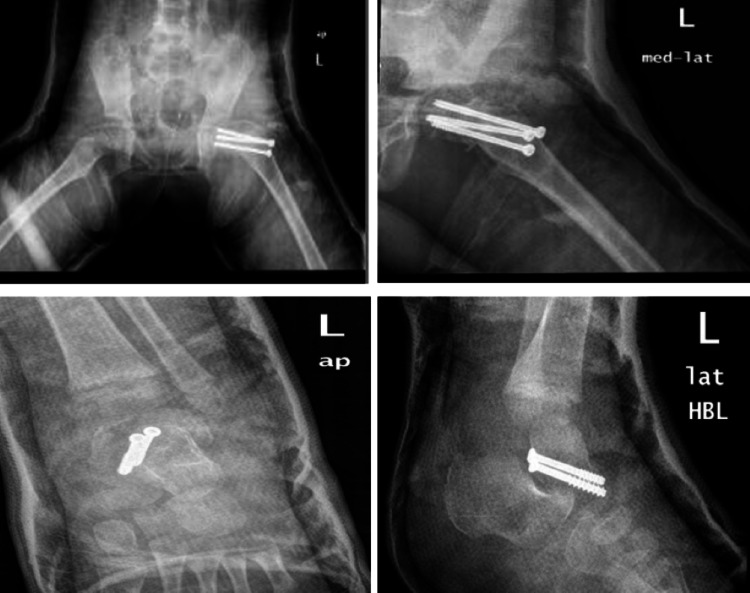
X-ray at two weeks follow-up post-operation

**Figure 6:**
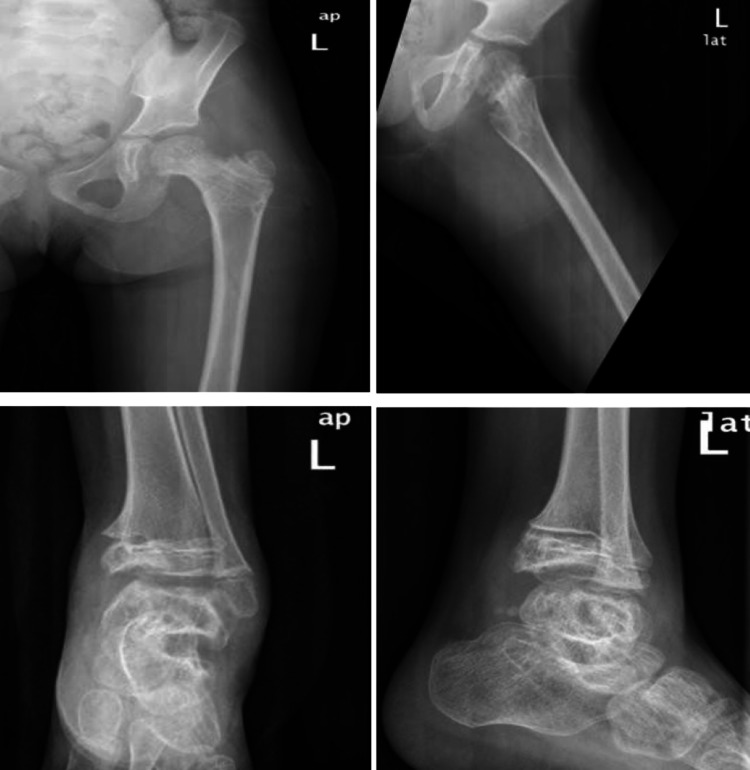
X-ray from a two-year follow-up with implant removal

The patient recovered well, tolerated the procedure, and the patient's mother was instructed by the medical team on the specifics of spica care. The patient was scheduled to be discharged two days after the surgery. Then, the patient returned for follow-up after two weeks with a foul smell from the spica (Figure 5). Thus, she was taken again to the operation room for a spica change and dressing under anesthesia. The patient was closely followed up by the outpatient department and her spica was removed after three months. The patient showed positive initial improvement with a physical therapy plan that aimed for mobilization on a wheelchair and full weight-bearing. One year post-surgical fixation, the patient presented to the clinic with a normal gait, no active complaint, and her x-ray showed complete healing. The implant removal was performed and the patient was followed up by the outpatient department for two years after the initial surgery with full recovery and no sign of avascular necrosis (AVN) (Figure 6).

## Discussion

**Figure 7:**
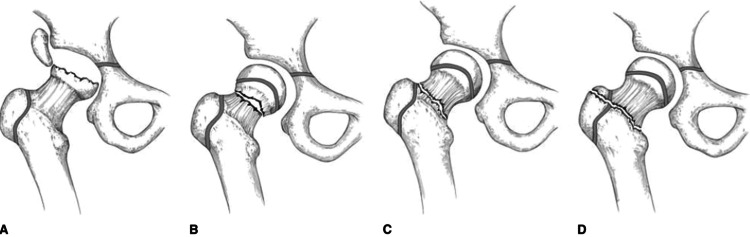
Illustration of the Delbet classification of hip fractures in children and adolescents. (A) Type I, transphyseal fracture, with or without dislocation of the capital femoral epiphysis. (B) Type II, transcervical fracture. (C) Type III, cervicotrochanteric fracture. (D) Type IV, intertrochanteric fracture. Source: Ref. [19]

Accidental fractures are common injuries in childhood and yet represent significant danger factors to the lives of children. The incidence of bone fractures among children also increases with age. Multiple fractures are uncommon in children and usually result from high-energy trauma. The present case is a six-year-old girl presented with an uncommon site of fracture at femoral neck combined with displaced talus fracture due to fall from the second floor. As first described by Delbert (Figure 7) [9]. Pediatric hip fractures can be divided into four types that help determine operative versus non-operative treatment and predict the risk of avascular necrosis (AVN) of the femoral head. Type I is a transphyseal fracture, type II is a transcervical fracture (the most common type), while type III is a cervicotrochanteric fracture, and type IV is an extracapsular intertrochanteric fracture. These fractures are also divided into two types of with and without displacement [9,10]. The present case showed two types of femur fracture (type I and type III) and is as per Delbert classification associated with a 15 times higher risk of AVN when compared to other fracture types [11]. However, there are cases in the medical literature that are not fit to be classified according to the Delbet classification, such as transcervical fracture with a second fracture line extending from the primary fracture line to physis, resulting in the separation of about half of the physis from the epiphysis [12,13]. Multiple fractures are consistently linked to high-energy trauma, and the present case had a talus fracture combined with a femur fracture. The incidence for talus fracture accounts is very low of all pediatric fractures compared to 0.3% in adults [13,14]. The mechanism of injury can be attributed to the sudden dorsiflexion on a partially plantar flexed foot, and usually, this is due to falling from height [13,15]. In the context of treatment, multiple fractures among the pediatric population require rapid and precise intervention options that can vary based on age, Delbet classification, and displacement of the fracture [1,2]. Cast immobilization used to be the primary choice for managing childhood fractures and can be used alone or in combination with minimal internal fixation [10,17]. Femur fractures in pediatrics are usually treated by immediate spica casting or traction followed by casting. Non-surgical treatment with spica casting remains the initial method of treatment for all patients under the age of five years old [10]. However, children above that age threshold are now more commonly undergoing surgical intervention. Surgical treatment has reduced the burden of care for their families along with shortened hospital stays and decreased early disability rates and lowered disruption in families’ lives [10,18]. The herein presented case received an urgent surgical intervention of open reduction with internal fixation followed by spica casting for a period of three months.

## Conclusions

Proximal femur fractures, although rare in children, are at an equally high risk of complication as their adult counterparts, e.g., avascular necrosis. This report has shown that early surgical treatment with internal fixation followed by cast immobilization can significantly improve such additional injuries. However, immediate fixation improves the outcome and prevent soft tissue complication in talus fracture, although the risk of avascular necrosis and post-traumatic arthritis are common sequelae of both fractures and require long follow up to address such complications.
